# Detection of anti-*Trypanosoma cruzi* antibodies by chimeric antigens in chronic Chagas disease-individuals from endemic South American countries

**DOI:** 10.1371/journal.pone.0215623

**Published:** 2019-04-18

**Authors:** Rodrigo Pimenta Del-Rei, Leonardo Maia Leony, Paola Alejandra Fiorani Celedon, Nilson Ivo Tonin Zanchin, Mitermayer Galvão dos Reis, Yara de Miranda Gomes, Alejandro Gabriel Schijman, Silvia Andrea Longhi, Fred Luciano Neves Santos

**Affiliations:** 1 Faculty of Technology and Sciences of Bahia, Salvador, Bahia, Brazil; 2 Gonçalo Moniz Institute, Oswaldo Cruz Foundation, Salvador, Bahia, Brazil; 3 Molecular Biology Institute of Paraná, Curitiba, Paraná, Brazil; 4 Carlos Chagas Institute, Oswaldo Cruz Foundation, Curitiba, Paraná, Brazil; 5 Department of Pathology and Legal Medicine, Federal University of Bahia, Salvador, Bahia, Brazil; 6 Department of Epidemiology of Microbial Diseases, School of Public Health, Yale University, New Haven, Connecticut, United States of America; 7 Aggeu Magalhães Institute, Oswaldo Cruz Foundation, Recife, Pernambuco, Brazil; 8 Laboratory of Molecular Biology of Chagas Disease, Institute for Research on Genetic Engineering and Molecular Biology “Dr Héctor Torres”, Buenos Aires, Argentina; Instituto de Ciências Biológicas, Universidade Federal de Minas Gerais, BRAZIL

## Abstract

**Background:**

Laboratory diagnosis of chronic Chagas disease is a troubling factor due to lack of reference tests. The WHO suggests the use of two distinct commercial serological tests in parallel. The performance of commercial immunoassays might fluctuate depending on the antigenic matrices and the local strains of *T*. *cruzi* in different geographical settings. The use of antigenic matrices based on chimeric proteins can solve these limitations. Here, we evaluated the diagnostic performance of two chimeric *T*. *cruzi* antigens (IBMP-8.1 and -8.4) to diagnose chronic Chagas disease in individuals from endemic South American countries.

**Methodology/Principal findings:**

IBMP-8.1 and IBMP-8.4 chimeric antigens were expressed as soluble proteins in *E*. *coli* and purified using chromatography methods. Reactivity of IBMP-8.1 and IBMP-8.4 was assessed using an in-house ELISA with sera from 122 non-infected and 215 *T*. *cruzi*-infected individuals from Argentina, Bolivia, and Paraguay. Cut-off values were based on ROC curves and performance parameters were determined using a dichotomous approach. Area under the curve values were > 99.7% for both IBMP-8.1 and IBMP-8.4 antigens. IgG levels in *T*. *cruzi*-positive and negative samples were higher for IBMP-8.4 than IBMP-8.1. Both IBMP-8.1 and -8.4 were 100% specific, while IBMP-8.4 were 100% sensitive compared to IBMP-8.1 (95.3%). Admitting RI values of 1.0 ± 0.10 as the inconclusive interval, 6.2% of the samples tested using IBMP-8.1 and 2.1% using IBMP-8.4 fell inside the grey zone. Based on accuracy and diagnostic odds ratio values, IBMP-8.4 presented the best performance. Differences in sensitivity and IgG levels among the samples from Argentina, Bolivia, and Paraguay were not significant.

**Conclusions/Significance:**

Our findings showed a notable performance of IBMP-8.1 and -8.4 chimeric antigens in diagnosing chronic Chagas disease in individuals from endemic South American countries, confirming our hypothesis that these antigens could be used in geographical areas where distinct *T*. *cruzi* DTUs occur.

## Introduction

Chagas' disease (CD) is a life-threating zoonosis caused by the hemoflagellate protozoan *Trypanosoma cruzi*. The parasite is transmitted by contact with dejections of infected blood-sucking triatomine bugs, by tissue and organ transplantation, consumption of parasite-contaminated food or beverages, blood transfusion, and from mother-to-child during pregnancy [1]. Although *T*. *cruzi* was discovered over a century ago, it is still posing a substantial public health threat, bearing in mind that the vast majority of the affected individuals lack access to treatment and diagnosis. As a matter of fact, CD is considered an essential neglected disease in the Americas [2].

Laboratory diagnosis of the disease is a troubling factor because the reference test is based on direct visualization of motile trypomastigotes in a blood smear, restricting the useful window of the reference test to the first 4–8 weeks post-exposure, during the acute phase. Because of the low and intermittent parasitemia, the majority of CD diagnosis is performed during the chronic phase, employing immunoassays for indirect detection of specific anti-*T*. *cruzi* antibodies. Most immunoassays are based on antigenic matrices with particular *T*. *cruzi* amino acid sequences meant to bind to their complementary anti-*T*. *cruzi* antibodies [3]. Due to their simplicity, low cost, and efficiency, antibody-based assays are the diagnostic methods of choice in chronic CD. However, due in part to the substantial genetic variability of the pathogen, the performance of commercial immunoassays might fluctuate depending on the local strains of *T*. *cruzi* and the employed antigenic matrices in different geographical regions [4].

The use of antigenic matrices based on chimeric proteins can solve these limitations. Indeed, chimeras are composed of repetitive and conserved immunodominant amino acid fragments of several *T*. *cruzi*-proteins. Accordingly, the possibility of a false-negative result decreases due to the availability of several distinct epitopes to be recognized by specific anti-*T*. *cruzi* antibodies, despite the antigenic variability across *T*. *cruzi* DTUs. Owing to the increase of migration and shifts worldwide, gradually favoring the spreading of infected people in non-endemic areas and transforming the disease into a global health alarm [5–7], the development of new serological tests should be prioritized, mainly in North America, Europe, and Oceania countries. Recently, our group synthesized and investigated the performance of four chimeric proteins (IBMP-8.1, -8.2, -8.3, and -8.4) in detecting antibodies against *T*. *cruzi* in human serum [8–10]. We observed that the chimeric antigens maintained their performance despite the antigenic variability across Brazilian *T*. *cruzi* strains. In fact, samples from endemic (Bahia, Goiás, Minas Gerais, and Pernambuco States) and non-endemic Brazilian settings (Paraná State) were assayed, and the chimeras, mainly IBMP-8.1 and IBMP-8.4, rendered high accuracy values. Similar results were found when an international commercial panel composed of samples from the USA, Nicaragua, Mexico, and Argentina was also assayed, suggesting that the chimeras could be able to identify *T*. *cruzi*-positive individuals regardless their geographical origin [9]. To confirm our hypothesis, we evaluated the performance of IBMP-8.1 and IBMP-8.4 chimeras to diagnose Chagas disease in individuals from endemic South American countries. In this study, we preferred to assess the IBMP-8.1 and -8.4 antigens, because they had previously shown the highest performance values, among the evaluated antigens.

## Materials and methods

### Ethical statements

This investigation followed the tenets of the Declaration of Helsinki and Guidelines according to Resolution N°1480/11 of the “Ministerio de Salud” from Argentina and were approved by the Local Medical Ethics Committees named “Comité del Instituto Regional de Medicina de la Universidad Nacional del Nordeste (UNNE)”, Resistencia, Chaco; “IDACH (Chaco Aboriginal Institute)”; “Comité de Ética de Investigación en Salud (CIEIS)” y “Comité de Ética del Hospital Zonal de Añatuya", Añatuya, Santiago del Estero; and Committees of Ramos Mejía and Pirovano Hospitals from Buenos Aires. We employed samples from the biorepository of the Laboratory of Molecular Biology of Chagas Disease (Institute for Research on Genetic Engineering and Molecular Biology—INGEBI CONICET-UBA). In order to maintain confidentiality over patient information, the samples were anonymized so that the researchers do not have access to patient’s individual information avoiding the need for verbal or written consent.

### Recombinant chimeric protein acquisition

IBMP-8.1 and IBMP-8.4 were expressed as soluble proteins in *Escherichia coli*-Star (DE3) cells grown in Luria-Bertani medium supplemented with 0.5 mM isopropyl-β-D-1-thiogalactopyranoside (IPTG). Proteins were purified by both affinity and ion exchange chromatography, then quantified using a fluorometric assay. Plasmidial construct has already been described in Santos et al. [8].

### Clinical specimens

**Fig 1:**
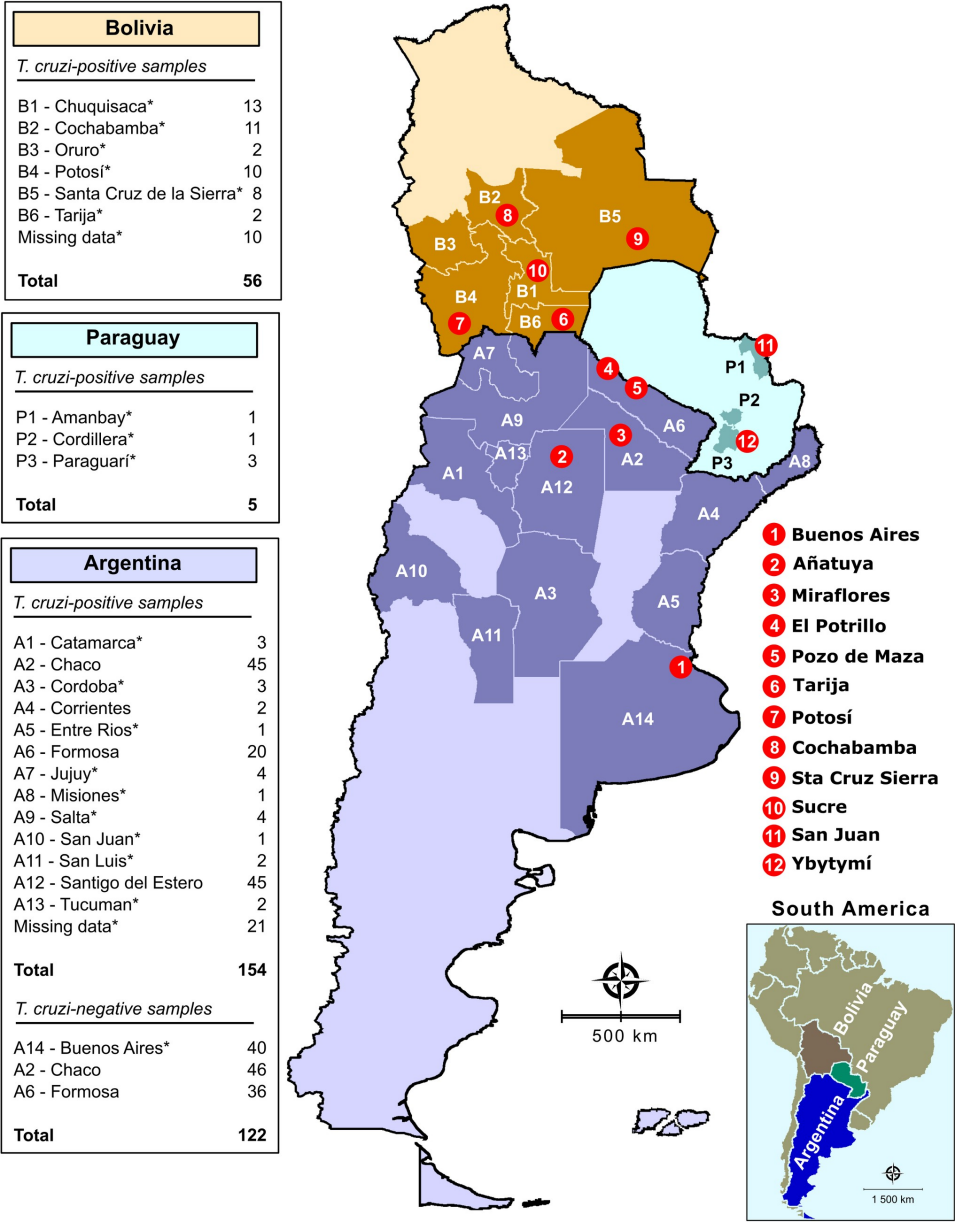
Geographical distribution in Argentina, Bolivia, and Paraguay of the samples. **Samples were collected from both *T*. *cruzi*-endemic and non-endemic areas.** The asterisk denotes the samples that were obtained in Buenos Aires health centers (the General Acute's Hospital Dr. Ignacio Pirovano and General Acute's Hospital JM Ramos Mejía) from patients who were born in the indicated endemic areas.

We used anonymized human sera obtained from INGEBI-CONICET serum bank, Buenos Aires, Argentina. Based on an expected error of 2%, sensitivity and specificity of 99% and a 95% confidence interval, the minimum sample was 96 sera from non-infected and 96 from *T*. *cruzi*-infected individuals. We included sera from 122 non-infected and 215 *T*. *cruzi*-infected individuals from rural endemic localities from 13 Argentine Provinces (Catamarca, Chaco, Cordoba, Corrientes, Entre Rios, Formosa, Jujuy, Misiones, Salta, San Juan, San Luis, Santiago del Estero, and Tucuman), from 6 Bolivian Departments (Chuquisaca, Cochabamba, Oruro, Potosí, Santa Cruz de la Sierra, and Tarija), and from three Departments in Paraguay (Amambay, Cordillera, and Paraguarí). Information about the city of origin was recovered only for some samples, such as those from Formosa, Chaco, Santiago del Estero, Chuquisaca, Cochabamba, Potosí, Santa Cruz de la Sierra, Tarija, Amambay, and Ybytymí. All other patients are from several localities in Bolivia, Paraguay, and Argentina and reside in Buenos Aires for medical attendance (Fig 1). The selection of clinical samples was based on positivity and negativity by two commercial serological tests (ELISA and/or indirect hemagglutination assays), according to World Health Organization advice [11]. Samples judged as inconclusive, or those that returned discordant results, were excluded. Each sample was assumed an identifier code in the laboratory to guarantee a blinded analysis. Digital map was obtained from the Brazilian Institute of Geography and Statistics (IBGE) cartographic database in shapefile (.shp), which was subsequently reformatted and analyzed using TerraView version 4.2, open source software freely available from the National Institute for Space Research (www.dpi.inpe.br/terraview).

### ELISA

Anti-*T*. *cruzi* serology was performed by ELISA according to previous reports [8,9]. Optical density was determined in a VersaMax microplate reader using a filter of 450 nm (Molecular Devices, San Jose, USA) and background values were subtracted from the measurement tests.

### Data analysis

**Fig 2:**
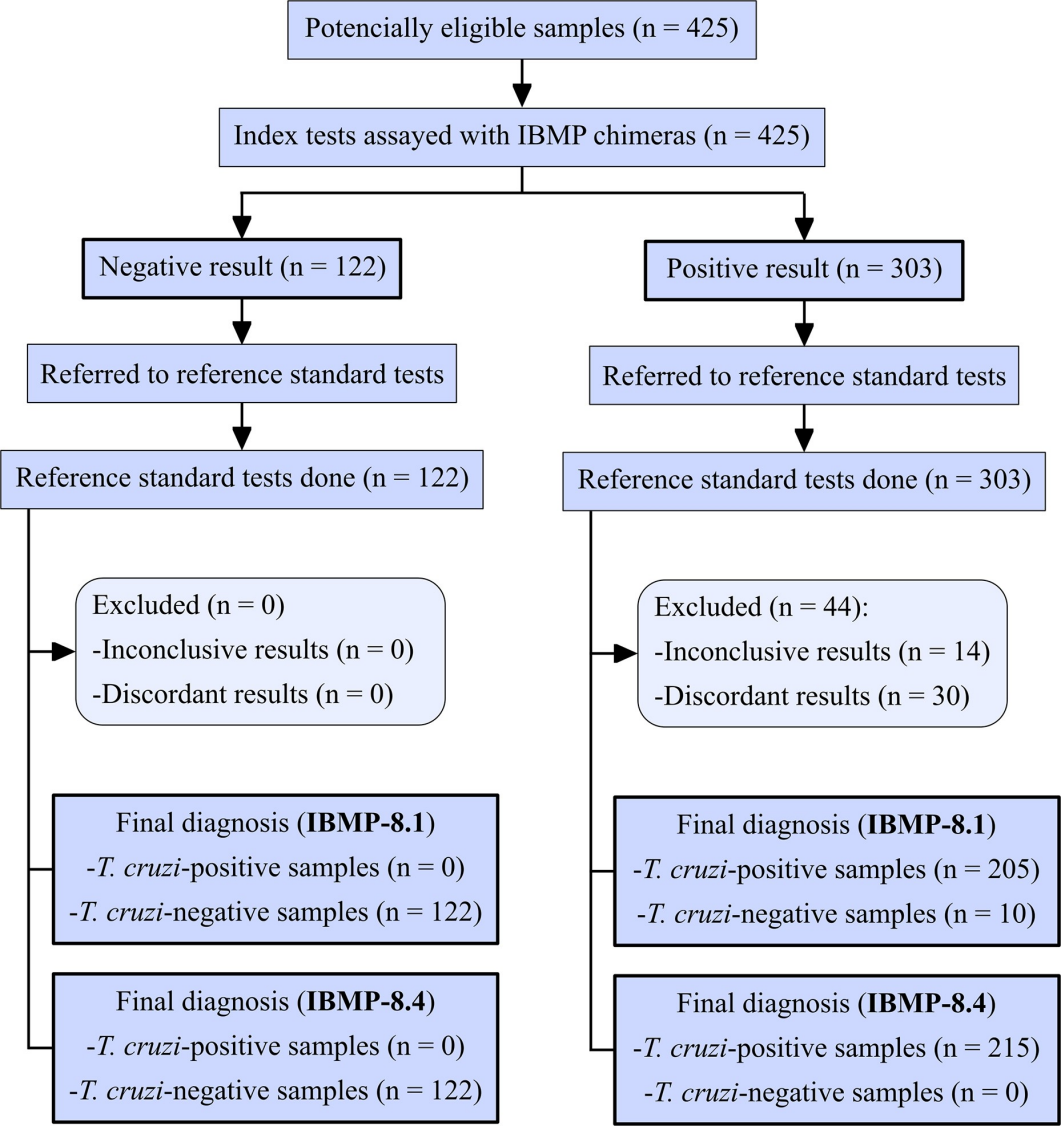
Flowchart depicting study design in conformity with the Standards for Reporting of Diagnostic Accuracy Studies (STARD) guidelines.

Data were encoded and analyzed using computer graphic software (GraphPad Prism version 7, San Diego, USA). Descriptive statistics were presented as geometric mean ± SD. Shapiro-Wilk test followed by Student’s t-test was used to the normality of datasets, and when the variance homogeneity assumption was not confirmed, the Wilcoxon signed-ranks test was used. All analyses were two-tailed and a p-value below 5% was considered significant (p < 0.05). Cut-off point analysis was used to identify the optimal value of OD that differentiates negative from positive samples. The threshold was defined by the largest distance from the diagonal line of the receiver operating characteristic curve (ROC). The results were expressed by plotting as an index that represents the ratio between the OD of the samples and the OD of the cut-off. This index is referred to as reactivity index (RI) and all results < 1.00 were considered negative. Samples were deemed inconclusive (or in grey zone) if the RI values fell into the undetermined zone, which was hypothesized as RI values of 1.0 ± 10%. ELISA performance was assessed using a dichotomous approach and compared with respect to sensitivity (Sen), specificity (Spe), accuracy (Acc), likelihood ratios (LR), and the diagnostic odds ratio (DOR). A flowchart (Fig 2) and a checklist (S1 Table) have been provided according to the Standards for Reporting of Diagnostic Accuracy Studies (STARD) guidelines [12].

## Results

**Fig 3:**
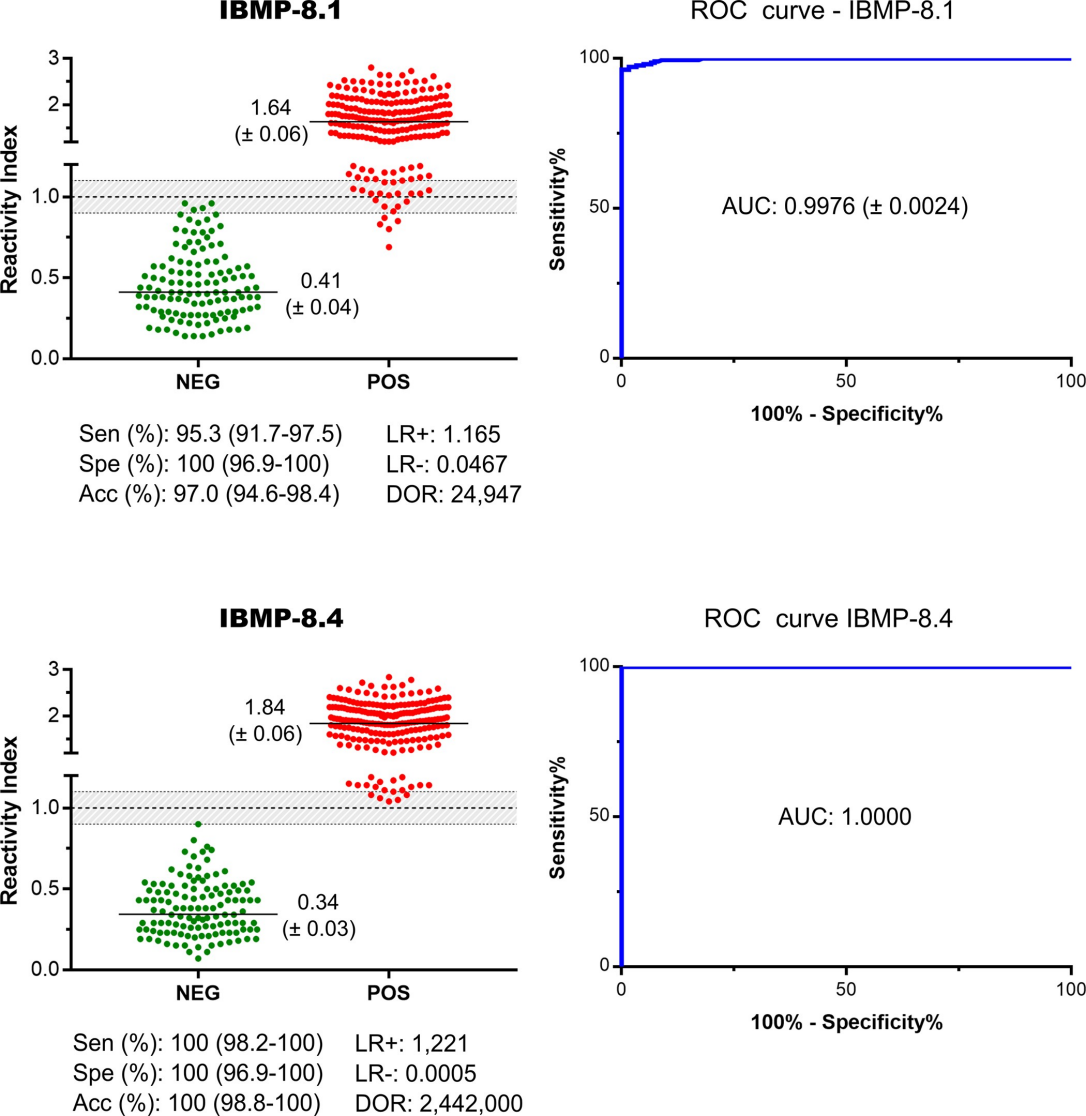
Reactivity index and performance parameters obtained with serum samples from *T*. *cruzi*-infected (POS) and non-infected (NEG) individuals. The cut-off value is reactivity index = 1.0 and the shadowed area represents the grey zone (RI = 1.0 ± 0.10). Horizontal lines and numbers for each group of results represent the geometric means (± 95% CI). AUC (Area Under Curve); Sen (Sensitivity); Spe (Specificity); Acc (Accuracy); LR (Likelihood Ratio); DOR (Diagnostic Odds Ratio).

The reactivity index (RI) distributions and assay performance parameters obtained for IBMP-8.1 and IBMP-8.4 chimeras are illustrated in Fig 3 (individual data points are available in the S2 Table). ROC curves were generated from 122 non-infected and 215 *T*. *cruzi*-infected individuals assayed by ELISA. Area under the curve (AUC) values were > 99.7%, demonstrating high overall diagnostic accuracy. IgG levels in *T*. *cruzi*-positive samples were variable, ranging from 1.64 for IBMP-8.1 to 1.84 for IBMP-8.4. For the panel of *T*. *cruzi*-positive samples, IBMP-8.4 chimera produced the highest sensitivity (100%). IBMP-8.1 showed 95.3% sensitivity with 10 cases classified as false-negatives. The differences between these values were statistically significant. Nonetheless, this difference was almost negligible, considering that the 95% CI values practically overlapped. Conversely, no false-positive results were obtained when *T*. *cruzi*-negative samples were assayed with IBMP-8.1 and IBMP-8.4, resulting in a specificity score of 100%.

Admitting RI values of 1.0 ± 0.10 as the inconclusive interval, we observed that four different samples (3.3%) and one (0.8%) *T*. *cruzi*-negative sample fell inside the grey zone employing IBMP-8.1 and IBMP-8.4 chimeras, respectively. Regarding *T*. *cruzi*-positive samples, we observed the following number of samples in the inconclusive interval: 17 (7.9%) assayed with IBMP-8.1 and 6 (2.8%) with IBMP-8.4. Overall analysis showed that 6.2% of the samples tested using IBMP-8.1 and 2.1% using IBMP-8.4 showed RI values falling in the grey zone (Fig 3). Of these, only four positive samples fell concomitantly inside the grey zone for IBMP-8.1 and IBMP8.4 chimeras.

IBMP-8.4 was found to most accurately diagnose Chagas disease (100%), followed by IBMP-8.1 (97.0%). Despite the statistical difference between these values, the 95% CI values practically overlapped. The test performance was summarized by the diagnostic odds ratio (DOR) value, which reached 24,947 for IBMP-8.1 (Fig 3). The IBMP-8.4 antigen showed an estimated DOR of 2,442.10^3^. Between the chimeric proteins tested, IBMP-8.4 presented the best performance, especially regarding its extremely high diagnostic odds ratio and AUC value.

**Fig 4:**
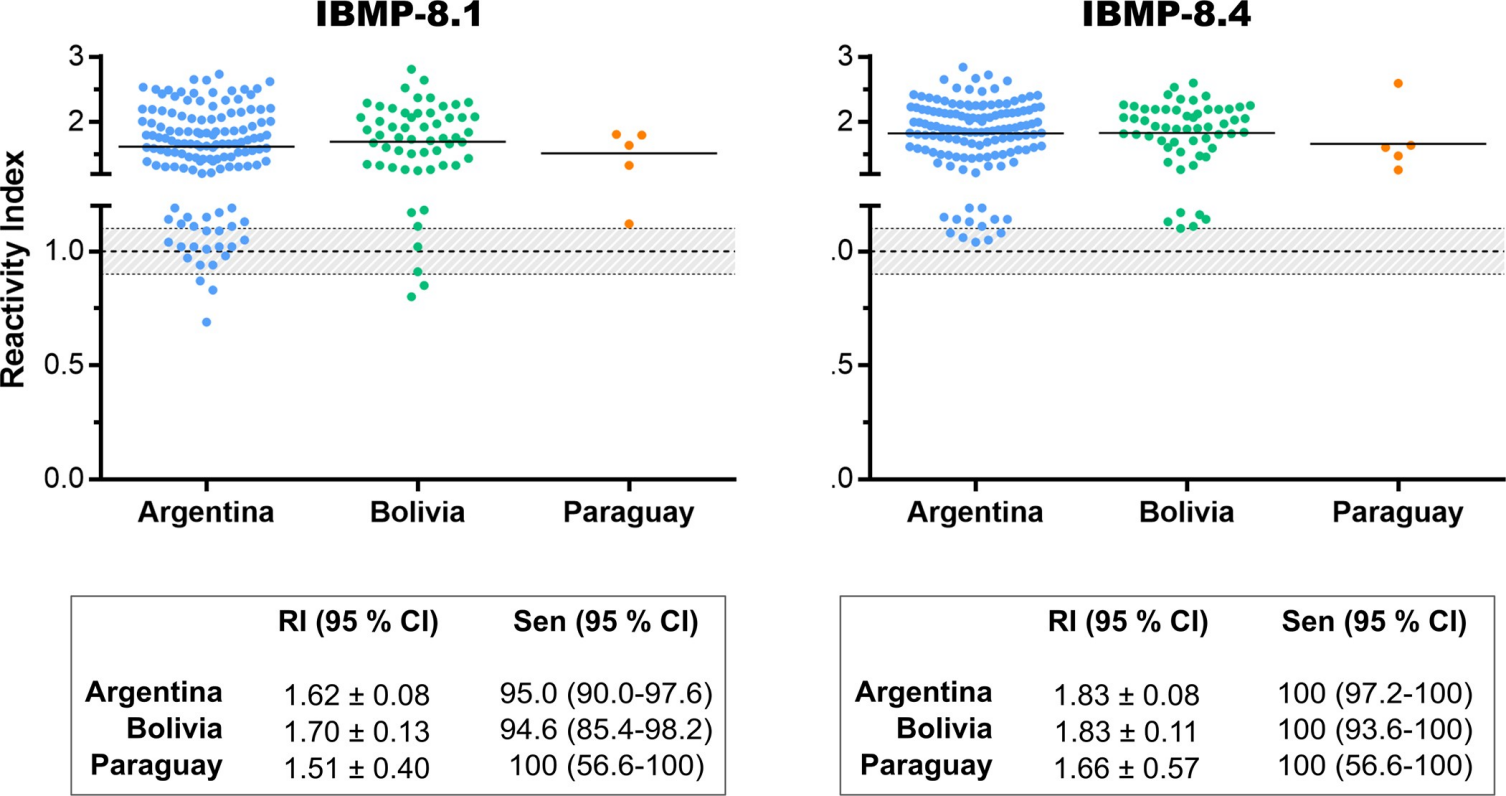
Reactivity Index for performance by country origin assessment. The cut-off value is reactivity index = 1.0 and the shadowed area represents the grey zone (RI = 1.0 ± 0.10). Horizontal lines and numbers for each group of results represent the geometric means (± 95% CI). RI (reactivity index); Sen (sensitivity); CI (confidence interval).

In order to evaluate the heterogeneity of recognition of IBMP-8.1 and IBMP-8.4 chimeras by anti-*T*. *cruzi* specific antibodies due to the expected regional genetic variability of parasite strains, RI and sensitivity values were compared using samples from *T*. *cruzi*-infected individuals residing in Argentina (n = 133), Bolivia (n = 56), and Paraguay (n = 5). We excluded 21 *T*. *cruzi*-positive samples from Buenos Aires city, not endemic for Chagas disease, due to the lack of information regarding the geographical precedence of the corresponding patients. Differences in sensitivity and RI signal among all geographical areas are not statistically significant (Fig 4; individual data points are available in the S3 Table).

## Discussion

The high genetic and phenotypic intraspecific diversity of *T*. *cruzi* is extensively recognized [13]; as it has been demonstrated using different biochemical, immunological and molecular markers [14,15]. Homologous pairs of chromosomes can vary in number and sizes between strains, as well as sequences and copy numbers of many genes, resulting in great genome plasticity [16]. Accordingly, the parasite has been grouped into seven evolutionary discrete typing units (DTUs) termed TcI–TcVI and Tcbat, with sub-classifications for regional strains in *clonets* and clones [4,17]. Regional parasite genetic variations have substantial implications in several features, such as epidemiological surveys [18], treatment response of *T*. *cruzi*-infected individuals [19], development of vaccines and drugs [20], prevalence of clinical forms and severity of manifestations [21,22], and even diagnosis [23]. Therefore, no single immunological test has sufficient performance to be used alone. In this way, we emphasize the need for the development of a diagnostic test able to identify chronic CD regardless of parasite genetic diversity. Here, two *T*. *cruzi* chimeric antigens were assayed with serum samples from patients residing in three endemic Latin America countries and returned accuracy values higher than 97%. The assays revealed a high diagnostic value. Indeed, the AUC values were greater than 99.7%, thereby showing an optimal discriminative power between chronic CD-positive and negative samples. These data are similar to previous results found by our group when samples from both Chagas disease endemic and non-endemic Brazilian settings were assayed either by ELISA [9] and liquid microarray tests [10].

The diagnostic sensitivity was lower for IBMP-8.1 compared to IBMP-8.4, with statistically significant differences between them. This difference is likely related to the antigenic composition of chimeras and genetic diversity of *T*. *cruzi*. In fact, IBMP-8.4 offers a vaster repertoire of epitopes compared to IBMP-8.1. Of note, IBMP-8.1 is composed of conserved and repetitive amino acid sequences of only three *T*. *cruzi* proteins (trans-sialidase, 60S ribosomal protein, and surface antigen 2) while IBMP-8.4 comprising epitopes from seven *T*. *cruzi* proteins (shed-acute-phase antigen, kinetoplastid membrane protein 11, trans-sialidase, flagellar repetitive antigen protein, surface antigen 2, 60S ribosomal protein, and microtubule-associated protein). According to previous studies performed in Brazil, a country where DTU TcII is predominant [4], the sensitivity displayed values higher than 97.4% and 99.1% for IBMP-8.1 and IBMP-8.4 chimeras, respectively [9,10]. Another study conducted on samples from Bolivian immigrants living in Barcelona/Spain showed a sensitivity of 99.4% for IBMP-8.1 and 99.1% for IBMP-8.4 [24]. It is important to note that TcV is the most frequent DTU found in Bolivia [4] and in Bolivian immigrants living in Barcelona [25]. These discrepancies may reflect weaker adaptive immune responses to parasite antigens between endemic populations [26].

Besides Bolivian samples, we also assayed samples from several endemic areas from Argentina and Paraguay, geographic areas where prevail TcV/TcVI and TcV/TcIII genotypes, respectively. However, no statistical difference was observed either with respect to sensitivity and reactivity index when these samples were stratified according to the country of origin. Although the number of Paraguayan samples is limited, we believe that the results can be repeated using a larger number of samples. The number of inconclusive results, based on a grey zone of 1.0 ± 10%, was higher for IBMP-8.1 compared to IBMP-8.4, which could be attributed to the antigenic structure or amino acid composition. Approximately 13.3% of positive samples fell in the inconclusive zone when assayed with IBMP-8.1 while only 5.5% presented this same behavior with IBMP-8.4. With respect to negative samples, the number of inconclusive results was low. In fact, four samples were inconclusive under IBMP-8.1 and only one under IBMP-8.4 analysis. No sample from Paraguay felt inside the grey zone. Overall, no significant coinciding inconclusive results were observed regarding IBMP-8.1 and IBMP8.4 assayed both positive and negative samples.

Other performance parameters were also considered here, such as positive and negative likelihood ratios and diagnostic odds ratio. Positive LR was higher than 1,000 for both IBMP-8.1 and IBMP-8.4 chimeras, indicating that a chronic Chagas disease carrier is approximately 1,000 times more likely to be diagnosed with this infection if evaluated with any of these antigens. Chagas disease-negative samples returned LR values lower than 5.10^−2^ for IBMP-8.1 and 5.10^−4^ for IBMP-8.4. There is an agreement that negative LRs below 0.1 and positives LRs above 10 contribute considerably to diagnosis [27]. DOR describes the probability of receiving a positive result for a person with infection, as opposed to someone who is non-infected [28]. It is a universal performance parameter that summarizes the diagnostic test accuracy. Here, we observed values greater than 24.10^3^, which are in accordance with previous results [9,10].

Our findings showed a notable performance of IBMP-8.1 and -8.4 chimeras in diagnosing chronic Chagas disease in individuals from endemic South American countries, confirming our hypothesis that these antigens could be used in geographical areas where distinct *T*. *cruzi* DTUs occur. The development of an accurate test for Chagas disease, regardless of *T*. *cruzi*-intrinsic antigenic variability, is of extreme importance within public health’s perspective, by simplifying diagnostic algorithms in relation to those presently used, making them more practical [29]. Furthermore, diagnostic costs may be reduced, due to the smaller number of samples that would need to be re-assayed with different diagnostic methods, or by repeating the test on another sample. However, further investigations are necessary to assess if these molecules maintain their performance in diagnosing the infection in individuals living in North and Central America or Northern region of South America, where Tc I prevails and Tc IV-infected cases have been detected.

## Supporting information

S1 Table STARD checklist. Standards for the Reporting of Diagnostic Accuracy Studies (STARD) checklist for reporting of studies of diagnostic accuracy.
(PDF)

S2 Table Reactivity Index for diagnostic performance assessment. (PDF)

S3 Table Reactivity Index for performance by country origin assessment. (PDF)
