# Supplementary material for: Detection of anti-Trypanosoma cruzi antibodies by chimeric antigens in chronic Chagas disease-individuals from endemic South American countries
Source: PLoS One. 2019 Apr 18;14(4):e0215623. doi: 10.1371/journal.pone.0215623 (PMC6472793; doi:10.1371/journal.pone.0215623)
Supplement: S2 Table — (PDF) [file pone.0215623.s002.pdf]

Samples	Reactivity Index	
	IBMP-8.1	IBMP-8.4
T. cruzi-negative_1	0.15	0.14
T. cruzi-negative_2	0.47	0.43
T. cruzi-negative_3	0.70	0.58
T. cruzi-negative_4	0.28	0.20
T. cruzi-negative_5	0.26	0.25
T. cruzi-negative_6	0.38	0.34
T. cruzi-negative_7	0.37	0.33
T. cruzi-negative_8	0.50	0.38
T. cruzi-negative_9	0.29	0.26
T. cruzi-negative_10	0.49	0.50
T. cruzi-negative_11	0.47	0.40
T. cruzi-negative_12	0.50	0.47
T. cruzi-negative_13	0.16	0.23
T. cruzi-negative_14	0.14	0.16
T. cruzi-negative_15	0.43	0.33
T. cruzi-negative_16	0.27	0.24
T. cruzi-negative_17	0.57	0.43
T. cruzi-negative_18	0.46	0.38
T. cruzi-negative_19	0.92	0.74
T. cruzi-negative_20	0.80	0.52
T. cruzi-negative_21	0.89	0.64
T. cruzi-negative_22	0.86	0.70
T. cruzi-negative_23	0.78	0.68
T. cruzi-negative_24	0.60	0.43
T. cruzi-negative_25	0.68	0.59
T. cruzi-negative_26	0.79	0.53
T. cruzi-negative_27	0.96	0.80
T. cruzi-negative_28	0.60	0.43
T. cruzi-negative_29	0.75	0.57
T. cruzi-negative_30	0.69	0.52
T. cruzi-negative_31	0.66	0.48
T. cruzi-negative_32	0.96	0.73
T. cruzi-negative_33	0.41	0.50
T. cruzi-negative_34	0.89	0.63
T. cruzi-negative_35	0.72	0.53
T. cruzi-negative_36	0.71	0.49
T. cruzi-negative_37	0.93	0.73
T. cruzi-negative_38	0.71	0.54
T. cruzi-negative_39	0.57	0.53
T. cruzi-negative_40	0.54	0.44
T. cruzi-negative_41	0.86	0.59
T. cruzi-negative_42	0.72	0.52
T. cruzi-negative_43	0.56	0.45
T. cruzi-negative_44	0.82	0.62
T. cruzi-negative_45	0.52	0.45
T. cruzi-negative_46	0.63	0.54

Samples	Reactivity Index	
	IBMP-8.1	IBMP-8.4
T. cruzi-negative_47	0.62	0.43
T. cruzi-negative_48	0.14	0.21
T. cruzi-negative_49	0.53	0.48
T. cruzi-negative_50	0.40	0.49
T. cruzi-negative_51	0.79	0.61
T. cruzi-negative_52	0.46	0.50
T. cruzi-negative_53	0.19	0.25
T. cruzi-negative_54	0.24	0.28
T. cruzi-negative_55	0.59	0.55
T. cruzi-negative_56	0.40	0.32
T. cruzi-negative_57	0.34	0.28
T. cruzi-negative_58	0.37	0.34
T. cruzi-negative_59	0.44	0.76
T. cruzi-negative_60	0.27	0.29
T. cruzi-negative_61	0.23	0.23
T. cruzi-negative_62	0.18	0.25
T. cruzi-negative_63	0.28	0.29
T. cruzi-negative_64	0.18	0.21
T. cruzi-negative_65	0.32	0.48
T. cruzi-negative_66	0.41	0.43
T. cruzi-negative_67	0.54	0.55
T. cruzi-negative_68	0.32	0.31
T. cruzi-negative_69	0.42	0.47
T. cruzi-negative_70	0.27	0.36
T. cruzi-negative_71	0.78	0.90
T. cruzi-negative_72	0.22	0.27
T. cruzi-negative_73	0.18	0.38
T. cruzi-negative_74	0.38	0.43
T. cruzi-negative_75	0.29	0.29
T. cruzi-negative_76	0.30	0.32
T. cruzi-negative_77	0.47	0.43
T. cruzi-negative_78	0.41	0.46
T. cruzi-negative_79	0.14	0.29
T. cruzi-negative_80	0.51	0.52
T. cruzi-negative_81	0.44	0.34
T. cruzi-negative_82	0.24	0.18
T. cruzi-negative_83	0.37	0.29
T. cruzi-negative_84	0.40	0.30
T. cruzi-negative_85	0.21	0.15
T. cruzi-negative_86	0.51	0.27
T. cruzi-negative_87	0.38	0.26
T. cruzi-negative_88	0.60	0.42
T. cruzi-negative_89	0.44	0.27
T. cruzi-negative_90	0.36	0.23
T. cruzi-negative_91	0.58	0.43
T. cruzi-negative_92	0.31	0.19

Samples	Reactivity Index	
	IBMP-8.1	IBMP-8.4
T. cruzi-negative_93	0.39	0.25
T. cruzi-negative_94	0.26	0.23
T. cruzi-negative_95	0.29	0.19
T. cruzi-negative_96	0.52	0.38
T. cruzi-negative_97	0.84	0.49
T. cruzi-negative_98	0.25	0.19
T. cruzi-negative_99	0.49	0.37
T. cruzi-negative_100	0.18	0.15
T. cruzi-negative_101	0.53	0.36
T. cruzi-negative_102	0.17	0.07
T. cruzi-negative_103	0.57	0.38
T. cruzi-negative_104	0.41	0.23
T. cruzi-negative_105	0.27	0.15
T. cruzi-negative_106	0.38	0.29
T. cruzi-negative_107	0.38	0.22
T. cruzi-negative_108	0.34	0.19
T. cruzi-negative_109	0.29	0.17
T. cruzi-negative_110	0.53	0.29
T. cruzi-negative_111	0.51	0.29
T. cruzi-negative_112	0.32	0.18
T. cruzi-negative_113	0.37	0.21
T. cruzi-negative_114	0.30	0.16
T. cruzi-negative_115	0.22	0.11
T. cruzi-negative_116	0.38	0.25
T. cruzi-negative_117	0.19	0.11
T. cruzi-negative_118	0.36	0.23
T. cruzi-negative_119	0.35	0.38
T. cruzi-negative_120	0.47	0.58
T. cruzi-negative_121	0.27	0.22
T. cruzi-negative_122	0.41	0.32
T. cruzi-positive_1	1.14	1.04
T. cruzi-positive_2	2.50	1.61
T. cruzi-positive_3	2.65	1.79
T. cruzi-positive_4	1.67	1.53
T. cruzi-positive_5	1.62	1.75
T. cruzi-positive_6	1.80	1.84
T. cruzi-positive_7	1.58	1.37
T. cruzi-positive_8	2.33	2.22
T. cruzi-positive_9	1.05	1.14
T. cruzi-positive_10	1.17	1.14
T. cruzi-positive_11	1.33	1.06
T. cruzi-positive_12	1.75	1.80
T. cruzi-positive_13	1.57	1.67
T. cruzi-positive_14	1.82	1.77
T. cruzi-positive_15	1.66	1.97
T. cruzi-positive_16	2.49	2.47

Samples	Reactivity Index	
	IBMP-8.1	IBMP-8.4
T. cruzi-positive_17	1.85	2.06
T. cruzi-positive_18	1.46	1.81
T. cruzi-positive_19	1.11	1.32
T. cruzi-positive_20	1.40	1.62
T. cruzi-positive_21	1.15	1.22
T. cruzi-positive_22	1.84	1.90
T. cruzi-positive_23	0.98	1.11
T. cruzi-positive_24	1.80	1.87
T. cruzi-positive_25	2.03	1.88
T. cruzi-positive_26	2.01	2.15
T. cruzi-positive_27	1.98	2.01
T. cruzi-positive_28	1.04	1.44
T. cruzi-positive_29	1.51	1.64
T. cruzi-positive_30	0.94	1.14
T. cruzi-positive_31	2.15	2.00
T. cruzi-positive_32	1.02	1.19
T. cruzi-positive_33	1.46	1.45
T. cruzi-positive_34	2.32	2.12
T. cruzi-positive_35	2.53	2.28
T. cruzi-positive_36	1.53	1.38
T. cruzi-positive_37	2.19	2.39
T. cruzi-positive_38	2.44	2.26
T. cruzi-positive_39	1.31	1.90
T. cruzi-positive_40	1.34	1.70
T. cruzi-positive_41	1.28	1.49
T. cruzi-positive_42	1.71	1.85
T. cruzi-positive_43	1.67	1.83
T. cruzi-positive_44	1.79	2.12
T. cruzi-positive_45	2.19	2.17
T. cruzi-positive_46	1.40	1.89
T. cruzi-positive_47	1.86	2.07
T. cruzi-positive_48	0.83	1.13
T. cruzi-positive_49	1.19	1.49
T. cruzi-positive_50	2.09	2.18
T. cruzi-positive_51	2.48	2.63
T. cruzi-positive_52	2.42	2.22
T. cruzi-positive_53	1.85	1.94
T. cruzi-positive_54	1.21	1.50
T. cruzi-positive_55	1.11	1.32
T. cruzi-positive_56	2.01	2.67
T. cruzi-positive_57	1.69	2.13
T. cruzi-positive_58	0.91	1.17
T. cruzi-positive_59	1.31	1.72
T. cruzi-positive_60	1.33	2.53
T. cruzi-positive_61	1.88	2.25
T. cruzi-positive_62	1.67	2.19

Samples	Reactivity Index	
	IBMP-8.1	IBMP-8.4
T. cruzi-positive_63	0.69	1.27
T. cruzi-positive_64	1.25	2.02
T. cruzi-positive_65	1.76	2.19
T. cruzi-positive_66	1.19	1.59
T. cruzi-positive_67	1.79	2.06
T. cruzi-positive_68	1.43	1.89
T. cruzi-positive_69	1.76	2.38
T. cruzi-positive_70	1.32	1.88
T. cruzi-positive_71	1.60	2.24
T. cruzi-positive_72	1.12	1.86
T. cruzi-positive_73	2.05	2.52
T. cruzi-positive_74	1.61	2.24
T. cruzi-positive_75	1.22	1.94
T. cruzi-positive_76	1.09	1.84
T. cruzi-positive_77	1.21	1.69
T. cruzi-positive_78	1.68	2.07
T. cruzi-positive_79	1.69	2.19
T. cruzi-positive_80	1.75	2.35
T. cruzi-positive_81	1.54	1.84
T. cruzi-positive_82	1.92	2.47
T. cruzi-positive_83	1.02	1.69
T. cruzi-positive_84	1.80	2.12
T. cruzi-positive_85	1.66	2.39
T. cruzi-positive_86	1.02	2.02
T. cruzi-positive_87	1.93	2.51
T. cruzi-positive_88	0.87	1.19
T. cruzi-positive_89	1.88	2.16
T. cruzi-positive_90	2.12	2.78
T. cruzi-positive_91	1.92	2.32
T. cruzi-positive_92	1.10	1.21
T. cruzi-positive_93	2.06	2.09
T. cruzi-positive_94	1.83	1.93
T. cruzi-positive_95	1.33	1.48
T. cruzi-positive_96	1.80	2.35
T. cruzi-positive_97	1.66	2.60
T. cruzi-positive_98	1.04	1.83
T. cruzi-positive_99	1.73	2.18
T. cruzi-positive_100	1.80	2.59
T. cruzi-positive_101	1.76	2.41
T. cruzi-positive_102	1.66	2.05
T. cruzi-positive_103	2.05	2.49
T. cruzi-positive_104	1.43	2.09
T. cruzi-positive_105	2.04	2.84
T. cruzi-positive_106	1.91	2.33
T. cruzi-positive_107	1.21	1.91
T. cruzi-positive_108	1.27	1.89

Samples	Reactivity Index	
	IBMP-8.1	IBMP-8.4
T. cruzi-positive_109	1.16	1.63
T. cruzi-positive_110	1.33	1.97
T. cruzi-positive_111	1.39	2.37
T. cruzi-positive_112	1.70	2.18
T. cruzi-positive_113	2.02	2.63
T. cruzi-positive_114	1.61	2.10
T. cruzi-positive_115	1.15	1.75
T. cruzi-positive_116	1.50	2.57
T. cruzi-positive_117	1.76	2.65
T. cruzi-positive_118	1.84	1.91
T. cruzi-positive_119	1.73	1.82
T. cruzi-positive_120	1.84	2.03
T. cruzi-positive_121	1.31	1.57
T. cruzi-positive_122	1.31	1.57
T. cruzi-positive_123	2.15	2.30
T. cruzi-positive_124	1.75	2.25
T. cruzi-positive_125	0.80	1.16
T. cruzi-positive_126	1.64	1.86
T. cruzi-positive_127	2.43	2.42
T. cruzi-positive_128	1.29	1.57
T. cruzi-positive_129	1.13	1.63
T. cruzi-positive_130	1.54	1.97
T. cruzi-positive_131	1.17	1.11
T. cruzi-positive_132	1.99	1.91
T. cruzi-positive_133	2.08	2.05
T. cruzi-positive_134	2.27	2.40
T. cruzi-positive_135	2.24	2.13
T. cruzi-positive_136	2.07	1.82
T. cruzi-positive_137	2.29	2.20
T. cruzi-positive_138	1.76	1.61
T. cruzi-positive_139	1.63	1.73
T. cruzi-positive_140	1.02	1.08
T. cruzi-positive_141	2.37	2.20
T. cruzi-positive_142	1.59	1.46
T. cruzi-positive_143	2.21	1.93
T. cruzi-positive_144	1.09	1.15
T. cruzi-positive_145	2.62	2.26
T. cruzi-positive_146	2.46	2.26
T. cruzi-positive_147	2.73	2.28
T. cruzi-positive_148	2.64	2.42
T. cruzi-positive_149	1.44	1.75
T. cruzi-positive_150	1.81	1.64
T. cruzi-positive_151	0.85	1.10
T. cruzi-positive_152	2.35	2.24
T. cruzi-positive_153	2.24	2.26
T. cruzi-positive_154	1.65	2.36

Samples	Reactivity Index	
	IBMP-8.1	IBMP-8.4
T. cruzi-positive_155	2.39	2.07
T. cruzi-positive_156	1.97	2.23
T. cruzi-positive_157	2.14	2.19
T. cruzi-positive_158	2.17	2.23
T. cruzi-positive_159	2.21	2.07
T. cruzi-positive_160	2.51	2.50
T. cruzi-positive_161	1.45	1.81
T. cruzi-positive_162	2.45	2.72
T. cruzi-positive_163	1.56	1.54
T. cruzi-positive_164	1.85	2.00
T. cruzi-positive_165	2.19	2.23
T. cruzi-positive_166	2.30	2.18
T. cruzi-positive_167	1.55	1.95
T. cruzi-positive_168	2.00	2.10
T. cruzi-positive_169	1.01	1.45
T. cruzi-positive_170	1.35	1.48
T. cruzi-positive_171	2.14	2.28
T. cruzi-positive_172	2.14	1.81
T. cruzi-positive_173	1.56	1.46
T. cruzi-positive_174	1.43	1.50
T. cruzi-positive_175	2.14	1.80
T. cruzi-positive_176	1.61	1.85
T. cruzi-positive_177	2.24	1.91
T. cruzi-positive_178	1.18	1.38
T. cruzi-positive_179	2.20	2.32
T. cruzi-positive_180	2.07	1.71
T. cruzi-positive_181	2.37	2.07
T. cruzi-positive_182	1.64	1.61
T. cruzi-positive_183	1.72	1.99
T. cruzi-positive_184	2.07	2.12
T. cruzi-positive_185	0.94	1.08
T. cruzi-positive_186	2.07	2.19
T. cruzi-positive_187	1.40	1.76
T. cruzi-positive_188	2.81	1.89
T. cruzi-positive_189	2.13	1.81
T. cruzi-positive_190	1.26	1.32
T. cruzi-positive_191	1.56	1.78
T. cruzi-positive_192	2.33	2.18
T. cruzi-positive_193	1.92	1.92
T. cruzi-positive_194	1.12	1.26
T. cruzi-positive_195	1.53	1.53
T. cruzi-positive_196	1.50	1.41
T. cruzi-positive_197	1.02	1.13
T. cruzi-positive_198	2.01	2.05
T. cruzi-positive_199	1.27	1.33
T. cruzi-positive_200	1.11	1.14

Samples	Reactivity Index	
	IBMP-8.1	IBMP-8.4
T. cruzi-positive_201	1.84	1.69
T. cruzi-positive_202	2.50	2.27
T. cruzi-positive_203	1.71	1.84
T. cruzi-positive_204	1.51	1.60
T. cruzi-positive_205	1.65	1.78
T. cruzi-positive_206	1.92	1.90
T. cruzi-positive_207	1.86	2.14
T. cruzi-positive_208	2.64	2.15
T. cruzi-positive_209	2.52	2.03
T. cruzi-positive_210	1.85	1.95
T. cruzi-positive_211	1.46	1.83
T. cruzi-positive_212	1.53	1.46
T. cruzi-positive_213	1.30	1.27
T. cruzi-positive_214	1.33	1.71
T. cruzi-positive_215	0.97	1.05
