# Supplementary material for: Detection of anti-Trypanosoma cruzi antibodies by chimeric antigens in chronic Chagas disease-individuals from endemic South American countries
Source: PLoS One. 2019 Apr 18;14(4):e0215623. doi: 10.1371/journal.pone.0215623 (PMC6472793; doi:10.1371/journal.pone.0215623)
Supplement: S3 Table — (PDF) [file pone.0215623.s003.pdf]

Samples	Country of origin	Reactivity Index	
		IBMP-8.1	IBMP-8.4
T. cruzi-positive_1	Argentina	1.14	1.04
T. cruzi-positive_2	Argentina	2.50	1.61
T. cruzi-positive_3	Argentina	2.65	1.79
T. cruzi-positive_4	Argentina	1.67	1.53
T. cruzi-positive_5	Argentina	1.62	1.75
T. cruzi-positive_6	Argentina	1.80	1.84
T. cruzi-positive_7	Argentina	1.58	1.37
T. cruzi-positive_8	Argentina	2.33	2.22
T. cruzi-positive_9	Argentina	1.05	1.14
T. cruzi-positive_10	Argentina	1.17	1.14
T. cruzi-positive_11	Argentina	1.33	1.06
T. cruzi-positive_12	Argentina	1.75	1.80
T. cruzi-positive_13	Argentina	1.57	1.67
T. cruzi-positive_14	Argentina	1.82	1.77
T. cruzi-positive_15	Argentina	1.66	1.97
T. cruzi-positive_16	Argentina	2.49	2.47
T. cruzi-positive_17	Argentina	1.85	2.06
T. cruzi-positive_18	Argentina	1.46	1.81
T. cruzi-positive_19	Argentina	1.11	1.32
T. cruzi-positive_20	Argentina	1.40	1.62
T. cruzi-positive_21	Argentina	1.15	1.22
T. cruzi-positive_22	Argentina	1.84	1.90
T. cruzi-positive_23	Argentina	0.98	1.11
T. cruzi-positive_24	Argentina	1.80	1.87
T. cruzi-positive_25	Argentina	2.03	1.88
T. cruzi-positive_26	Argentina	2.01	2.15
T. cruzi-positive_27	Argentina	1.98	2.01
T. cruzi-positive_28	Argentina	1.04	1.44
T. cruzi-positive_29	Argentina	1.51	1.64
T. cruzi-positive_30	Argentina	0.94	1.14
T. cruzi-positive_31	Argentina	2.15	2.00
T. cruzi-positive_32	Argentina	1.02	1.19
T. cruzi-positive_33	Argentina	1.46	1.45
T. cruzi-positive_34	Argentina	2.32	2.12
T. cruzi-positive_35	Argentina	2.53	2.28
T. cruzi-positive_36	Argentina	1.53	1.38
T. cruzi-positive_37	Argentina	2.19	2.39
T. cruzi-positive_38	Argentina	2.44	2.26
T. cruzi-positive_39	Argentina	1.31	1.90
T. cruzi-positive_40	Argentina	1.34	1.70
T. cruzi-positive_41	Argentina	1.28	1.49
T. cruzi-positive_42	Argentina	1.71	1.85
T. cruzi-positive_43	Argentina	1.67	1.83
T. cruzi-positive_44	Argentina	1.79	2.12
T. cruzi-positive_45	Argentina	2.19	2.17
T. cruzi-positive_46	Argentina	1.40	1.89

Samples	Country of origin	Reactivity Index	
		IBMP-8.1	IBMP-8.4
T. cruzi-positive_47	Argentina	1.86	2.07
T. cruzi-positive_48	Argentina	0.83	1.13
T. cruzi-positive_49	Argentina	1.19	1.49
T. cruzi-positive_50	Argentina	2.09	2.18
T. cruzi-positive_51	Argentina	2.48	2.63
T. cruzi-positive_52	Argentina	2.42	2.22
T. cruzi-positive_53	Argentina	1.85	1.94
T. cruzi-positive_54	Argentina	1.21	1.50
T. cruzi-positive_55	Argentina	1.11	1.32
T. cruzi-positive_56	Argentina	2.01	2.67
T. cruzi-positive_57	Argentina	1.69	2.13
T. cruzi-positive_58	Argentina	1.31	1.72
T. cruzi-positive_59	Argentina	1.67	2.19
T. cruzi-positive_60	Argentina	0.69	1.27
T. cruzi-positive_61	Argentina	1.19	1.59
T. cruzi-positive_62	Argentina	1.43	1.89
T. cruzi-positive_63	Argentina	1.32	1.88
T. cruzi-positive_64	Argentina	1.60	2.24
T. cruzi-positive_65	Argentina	1.12	1.86
T. cruzi-positive_66	Argentina	2.05	2.52
T. cruzi-positive_67	Argentina	1.22	1.94
T. cruzi-positive_68	Argentina	1.09	1.84
T. cruzi-positive_69	Argentina	1.54	1.84
T. cruzi-positive_70	Argentina	1.02	1.69
T. cruzi-positive_71	Argentina	1.66	2.39
T. cruzi-positive_72	Argentina	1.02	2.02
T. cruzi-positive_73	Argentina	1.93	2.51
T. cruzi-positive_74	Argentina	0.87	1.19
T. cruzi-positive_75	Argentina	1.88	2.16
T. cruzi-positive_76	Argentina	1.92	2.32
T. cruzi-positive_77	Argentina	1.83	1.93
T. cruzi-positive_78	Argentina	1.80	2.35
T. cruzi-positive_79	Argentina	1.76	2.41
T. cruzi-positive_80	Argentina	1.66	2.05
T. cruzi-positive_81	Argentina	1.43	2.09
T. cruzi-positive_82	Argentina	2.04	2.84
T. cruzi-positive_83	Argentina	1.39	2.37
T. cruzi-positive_84	Argentina	1.76	2.65
T. cruzi-positive_85	Argentina	1.31	1.57
T. cruzi-positive_86	Argentina	1.75	2.25
T. cruzi-positive_87	Argentina	1.61	2.10
T. cruzi-positive_88	Argentina	1.56	1.46
T. cruzi-positive_89	Argentina	2.20	2.32
T. cruzi-positive_90	Argentina	1.45	1.81
T. cruzi-positive_91	Argentina	2.39	2.07
T. cruzi-positive_92	Argentina	2.51	2.50

Samples	Country of origin	Reactivity Index	
		IBMP-8.1	IBMP-8.4
T. cruzi-positive_93	Argentina	1.85	1.95
T. cruzi-positive_94	Argentina	1.46	1.83
T. cruzi-positive_95	Argentina	2.45	2.72
T. cruzi-positive_96	Argentina	2.14	1.80
T. cruzi-positive_97	Argentina	2.24	2.13
T. cruzi-positive_98	Argentina	1.99	1.91
T. cruzi-positive_99	Argentina	1.65	1.78
T. cruzi-positive_100	Argentina	1.15	1.75
T. cruzi-positive_101	Argentina	1.31	1.57
T. cruzi-positive_102	Argentina	2.43	2.42
T. cruzi-positive_103	Argentina	1.29	1.57
T. cruzi-positive_104	Argentina	1.63	1.73
T. cruzi-positive_105	Argentina	1.02	1.08
T. cruzi-positive_106	Argentina	1.09	1.15
T. cruzi-positive_107	Argentina	2.62	2.26
T. cruzi-positive_108	Argentina	2.73	2.28
T. cruzi-positive_109	Argentina	2.35	2.24
T. cruzi-positive_110	Argentina	1.65	2.36
T. cruzi-positive_111	Argentina	2.21	2.07
T. cruzi-positive_112	Argentina	2.19	2.23
T. cruzi-positive_113	Argentina	1.01	1.45
T. cruzi-positive_114	Argentina	2.14	2.28
T. cruzi-positive_115	Argentina	1.43	1.50
T. cruzi-positive_116	Argentina	1.72	1.99
T. cruzi-positive_117	Argentina	0.94	1.08
T. cruzi-positive_118	Argentina	1.53	1.53
T. cruzi-positive_119	Argentina	1.86	2.14
T. cruzi-positive_120	Argentina	2.64	2.15
T. cruzi-positive_121	Argentina	0.97	1.05
T. cruzi-positive_122	Argentina	1.40	1.76
T. cruzi-positive_123	Argentina	1.13	1.63
T. cruzi-positive_124	Argentina	1.54	1.97
T. cruzi-positive_125	Argentina	1.59	1.46
T. cruzi-positive_126	Argentina	2.46	2.26
T. cruzi-positive_127	Argentina	2.17	2.23
T. cruzi-positive_128	Argentina	1.61	1.85
T. cruzi-positive_129	Argentina	2.07	2.12
T. cruzi-positive_130	Argentina	1.26	1.32
T. cruzi-positive_131	Argentina	2.33	2.18
T. cruzi-positive_132	Argentina	2.50	2.27
T. cruzi-positive_133	Argentina	1.85	2.00
T. cruzi-positive_134	Bolivia	1.25	2.02
T. cruzi-positive_135	Bolivia	1.76	2.19
T. cruzi-positive_136	Bolivia	2.06	2.09
T. cruzi-positive_137	Bolivia	1.66	2.60
T. cruzi-positive_138	Bolivia	2.08	2.05

Samples	Country of origin	Reactivity Index	
		IBMP-8.1	IBMP-8.4
T. cruzi-positive_139	Bolivia	2.07	1.82
T. cruzi-positive_140	Bolivia	2.29	2.20
T. cruzi-positive_141	Bolivia	2.14	1.81
T. cruzi-positive_142	Bolivia	2.07	1.71
T. cruzi-positive_143	Bolivia	2.81	1.89
T. cruzi-positive_144	Bolivia	2.13	1.81
T. cruzi-positive_145	Bolivia	1.02	1.13
T. cruzi-positive_146	Bolivia	1.11	1.14
T. cruzi-positive_147	Bolivia	1.53	1.46
T. cruzi-positive_148	Bolivia	1.27	1.89
T. cruzi-positive_149	Bolivia	0.85	1.10
T. cruzi-positive_150	Bolivia	1.97	2.23
T. cruzi-positive_151	Bolivia	2.14	2.19
T. cruzi-positive_152	Bolivia	1.35	1.48
T. cruzi-positive_153	Bolivia	2.07	2.19
T. cruzi-positive_154	Bolivia	1.27	1.33
T. cruzi-positive_155	Bolivia	1.84	1.69
T. cruzi-positive_156	Bolivia	1.71	1.84
T. cruzi-positive_157	Bolivia	0.91	1.17
T. cruzi-positive_158	Bolivia	1.33	2.53
T. cruzi-positive_159	Bolivia	1.88	2.25
T. cruzi-positive_160	Bolivia	1.61	2.24
T. cruzi-positive_161	Bolivia	1.68	2.07
T. cruzi-positive_162	Bolivia	1.69	2.19
T. cruzi-positive_163	Bolivia	1.80	2.12
T. cruzi-positive_164	Bolivia	1.33	1.97
T. cruzi-positive_165	Bolivia	0.80	1.16
T. cruzi-positive_166	Bolivia	2.30	2.18
T. cruzi-positive_167	Bolivia	1.18	1.38
T. cruzi-positive_168	Bolivia	1.56	1.78
T. cruzi-positive_169	Bolivia	1.73	1.82
T. cruzi-positive_170	Bolivia	1.17	1.11
T. cruzi-positive_171	Bolivia	2.27	2.40
T. cruzi-positive_172	Bolivia	1.76	1.61
T. cruzi-positive_173	Bolivia	1.44	1.75
T. cruzi-positive_174	Bolivia	2.24	2.26
T. cruzi-positive_175	Bolivia	2.37	2.07
T. cruzi-positive_176	Bolivia	1.92	1.92
T. cruzi-positive_177	Bolivia	2.01	2.05
T. cruzi-positive_178	Bolivia	1.51	1.60
T. cruzi-positive_179	Bolivia	2.37	2.20
T. cruzi-positive_180	Bolivia	2.64	2.42
T. cruzi-positive_181	Bolivia	1.56	1.54
T. cruzi-positive_182	Bolivia	1.92	1.90
T. cruzi-positive_183	Bolivia	2.52	2.03
T. cruzi-positive_184	Bolivia	1.30	1.27

Samples	Country of origin	Reactivity Index	
		IBMP-8.1	IBMP-8.4
T. cruzi-positive_185	Bolivia	1.33	1.71
T. cruzi-positive_186	Bolivia	1.75	2.35
T. cruzi-positive_187	Bolivia	1.91	2.33
T. cruzi-positive_188	Bolivia	2.21	1.93
T. cruzi-positive_189	Bolivia	2.24	1.91
T. cruzi-positive_190	Paraguay	1.33	1.48
T. cruzi-positive_191	Paraguay	1.80	2.59
T. cruzi-positive_192	Paraguay	1.81	1.64
T. cruzi-positive_193	Paraguay	1.64	1.61
T. cruzi-positive_194	Paraguay	1.12	1.26
